# Characterization of Insulin and Glucagon Genes and Their Producing Endocrine Cells From Pygmy Sperm Whale (*Kogia breviceps*)

**DOI:** 10.3389/fendo.2020.00174

**Published:** 2020-03-31

**Authors:** Liyuan Zhao, Likun Wang, Reyilamu Aierken, Wei Wang, Xianyan Wang, Mingyu Li

**Affiliations:** ^1^Laboratory of Marine Biology and Ecology, Fujian Provincial Key Laboratory of Marine Ecological Conservation and Restoration, Third Institute of Oceanography, Ministry of Natural Resources, Xiamen, China; ^2^School of Pharmaceutical Sciences, Fujian Provincial Key Laboratory of Innovative Drug Target Research, Xiamen University, Xiamen, China; ^3^Department of Endocrinology, Xiang'an Hospital of Xiamen University, School of Medicine, Xiamen University, Xiamen, China

**Keywords:** pygmy sperm whale, insulin, glucagon, α cell, β cell

## Abstract

Insulin and glucagon are hormones secreted by pancreatic β and α cells, respectively, which together regulate glucose homeostasis. Dysregulation of insulin or glucagon can result in loss of blood glucose control, characterized by hyperglycemia or hypoglycemia. To better understand the endocrine physiology of cetaceans, we cloned and characterized the *insulin* and *glucagon* genes from pygmy sperm whale (*Kogia breviceps)*. We obtained the complete coding sequences of the *preproinsulin* and *preproglucagon* genes, which encodes the preproinsulin protein of 110 amino acid (aa) residues and encodes the preproglucagon protein of 179 aa residues, respectively. Sequence comparison and phylogenetic analyses demonstrate that protein structures were similar to other mammalian orthologs. Immunohistochemistry and immunofluorescence staining using insulin, glucagon, and somatostatin antibodies allowed analysis of pygmy sperm whale islet distribution, architecture, and composition. Our results showed the pygmy sperm whale islet was irregularly shaped and randomly distributed throughout the pancreas. The architecture of α, β, and δ cells of the pygmy sperm whale was similar to that of artiodactyls species. This is the first report about *insulin* and *glucagon* genes in cetaceans, which provides new information about the structural conservation of the insulin and glucagon genes. Furthermore, offers novel information on the properties of endocrine cells in cetacean for further studies.

## Introduction

The pancreatic islet is composed of five different types of endocrine cells, including insulin-secreting β cells and glucagon-secreting α cells, which together regulates glucose homeostasis (1). Insulin is secreted by β cells, primarily in response to elevated concentrations of blood glucose, which causes glucose uptake in peripheral tissues, and a conversion to glycogen (2). Glucagon is produced by α cells and acts to oppose the functions of insulin, which elevates the concentration of glucose in the blood by promoting gluconeogenesis and glycogenolysis in peripheral tissues, predominantly in the liver (3). Due to the clinical importance of their related diseases, such as diabetes, these two hormones have been studied extensively and in-depth, in both human and mammalian animal models.

In mammals, both insulin and glucagon are evolutionary well-conserved. However, although insulin and glucagon have also been widely studied in non-mammalian species, information on the genes, physiology, and evolution of cetaceans still remains unclear. Cetaceans, commonly known as whales, dolphins, and porpoises, are mammals that have secondarily adapted to the aquatic environment, and are important organisms for the understanding of genetic evolution (4, 5). Upon aquatic transition cetaceans have acquired many characteristics, for example, a thick subcutaneous fatty deposit, or blubber, that is considered to be a primary energy store and is 10-fold greater than that of other artiodactyls species, the closest living relatives of cetaceans (6, 7). It is poorly understood how insulin and glucagon contribute to this special energy store. Interestingly, in these aquatic mammals the glucose homeostasis are similar, in process, to humans. Studies have revealed that bottlenose dolphins (*Tursiops truncatus*) have sustained post-prandial hyperglycemia and hyperinsulinemia, dyslipidemia, and fatty liver disease, which is similar to human diabetes (8–10).

In order to establish the molecular basis for further studies, we identified the *preproinsulin* and *preproglucagon* genes from a cetacean, pygmy sperm whale (*Kogia breviceps*). First, we performed sequence alignment and phylogenetic analysis of both genes. Second, we characterized the cetacean pancreatic endocrine α cells and β cells by immunohistochemistry and immunofluorescence.

## Materials and Methods

### Animal and Tissue Collection

On June 27th, 2019, a carcass of toothed whale was found in Pingtan Island, Fujian province in China, and taken to the Third Institute of Oceanography, Ministry of Natural Resources, China for further species identification and anatomy specimen making. External morphological assessment of the carcass was measured before the autopsy. And, sex was determined anatomically by the analysis of reproductive organs morphology. Since this toothed whale carcass showed fresh smell, normal appearance and with several scrapes in the skin, as well as eyes clear (Supplemental Figure 1A), it was classified as Code 2, <24 h post-mortem (11). During necropsy, all organ systems were examined macroscopically. Several tissues were collected and frozen at −20°C. Dissections were performed using standard anatomical instruments at the Endangered Animal Center, Laboratory of Marine Biology and Ecology, Third Institute of Oceanography, Ministry of Natural Resources, Xiamen, China.

The pancreas of toothed whale was removed from the whale and fixed for 4 h in 4% paraformaldehyde-PBS at 4°C. Followed equilibrated pancreas in 30% sucrose overnight until the tissue settles to bottom at 4°C. Pancreas was then placed in cryomold containing OCT and Store at −80°C for further tissues analysis. For the mouse pancreas experiments, the pancreas of a 19 weeks adult C57BL/6 mouse was used as control. The tissue collection protocol followed the study as previously reported (12). All procedures have been approved by the Xiamen University Institutional Animal Care and Use Committee (Protocol XMULAC20160089, 10 March 2016).

### Genetic Species Identification

For species identification, three partial fragments of the mtDNA were amplified. A partial fragment of cytochrome c oxidase subunit 1 (*cox1*) gene was amplified and sequenced using primers Lco1ea: 5′ -TCGGCCATTTTACCTATGTTCATA-3′ and Hbcuem: 5′ -GGTGGCCGAAGAATCAGAATA-3′ (13). The partial cytochrome b (*cytb*) gene was amplified and sequenced using primers L14724: 5′-TGACTTGAARAACCAYCGTTG-3′ and H15387: 5′-GAATGGGATTATGTCTATGT-3′ (14). The D loop region was amplified and sequenced using primers Ce-CRF: 5′-GAATTCCCCGGTCTTGTAAACC-3′ and Ce-CRR: 5′-TCTCGAGATTTTCAGTGTCTTGCTTT-3′ (15). The PCR products were directly sequenced in both directions. After deleting the flanking and primer sequence and edited by BioEdit, *cox1, cytb*, and *D-loop* were 636, 574, and 938 bp in length, respectively.

### Molecular Cloning, Structural, and Phylogenetic Analysis of Insulin and Glucagon Genes

**Figure 1:**
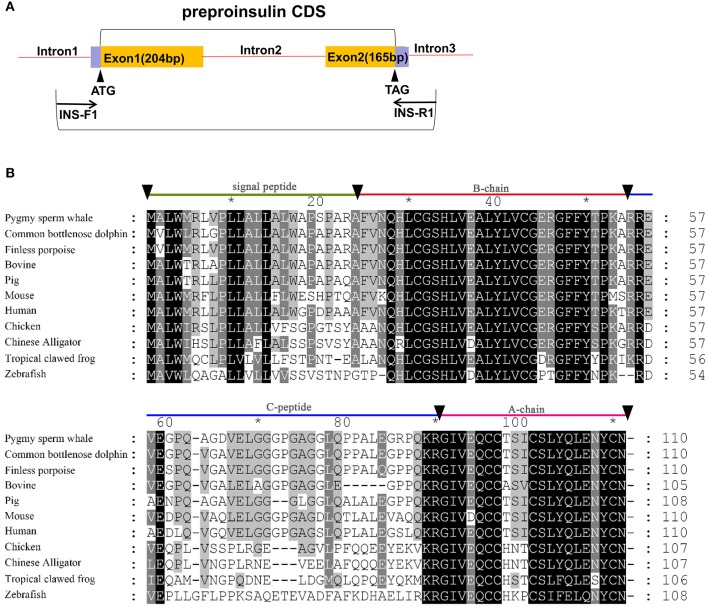
Identification of *preproinsulin* from pygmy sperm whale. **(A)** Schematic representation of the PCR strategy to clone *preproinsulin*. **(B)** Sequence comparison of vertebrate preproinsulin primary sequences. Sequence alignments were obtained with Genedoc by the Clustal method. Residues that are identical or conserved are shaded. The signal peptide (residues 1~24), B-chain (residues 25~54), C-peptide (55~89), and A-chain (residues 90~110) are labeled.

Based on the genomic comparison with several species *insulin* and *glucagon* loci, we designed the primers for both genes in the intron conserved region. We amplified *insulin* and *glucagon* genes from the genomic DNA sample. Primers were designed using the Primer Premier 5.0 software (Premier Biosoft International, Palo Alto, CA). For *insulin* gene, Primers are INS-F1: 5′-GGCGTGGGCTCCTCTCTT-3′ and INS-R1: 5′-AGGGCTCATTCAGGGGGTTT-3′ (Figure 1A). For *glucagon* gene, primers are GCG-F1:5′-CAGAGTGCTACACATCTTTTG-3′, GCG-R1:5′-GAGTAATGAATGAACGGGGAGT-3′; GCG-F2: 5′-AACGGTTTGGTATTCTTCACTC-3′, GCG-R2:5′-ATGTTTATCTTGGATTGTA-3′; GCG-F3: 5′-ACCTTTGCTTTCCCTTGATTC-3′, GCG-R3:5′ -CTCTGTTTATTCTCAAAAGTGTCCT-3′; GCG-F4: 5′-GTCTCAATAATAACTTCTGTG-3′, GCG-R4: 5′-GGCAATTACAACCTACCAAGA-3′ (**Figure 3A**). The PCR was performed using TaKaRa LA Taq with GC Buffer system (TaKaRa Bio Inc.) in a Veriti 96 well-thermal cycler (Applied Biosystem, CA, USA). PCR products were estimated by 1% agarose gel electrophoresis and sequenced in both directions at the Majorbio Sequencing (Shanghai, China).

Sequence identity analysis was performed among Human (*Homo sapiens*), Finless porpoise (*Neophocaena asiaeorientalis*), Common bottlenose dolphin (*Tursiops truncatus*), Pig (*Sus scrofa*), Chicken (*Gallus gallus*), Mouse (*Mus musculus*), Bovine (*Bos taurus)*, Chinese Alligator (*Alligator sinensis*), Tropical clawed frog (*Xenopus tropicalis*), Zebrafish (*Danio rerio*) using the CLUSTALW program (http://www.ebi.ac.uk/clustaw/). Phylogenetic analysis was done using full-length amino acid sequences containing 18 mammals (*K. breviceps, Homo sapiens, Mus musculus, Loxodonta africana, Oryctolagus cuniculus, Gorilla gorilla gorilla, Cavia porcellus, Oryctolagus cuniculus, Erinaceus europaeus, Canis lupus familiaris, Vulpes vulpes, Equus caballus, Tursiops truncatus, Neophocaena asiaeorientalis, Sus scrofa, Ovis aries, Bos taurus, Ornithorhynchus anatinus*), one Aves (*Gallus gallus*), one Reptilia (*Alligator sinensis*), one Amphibia (*Xenopus tropicalis*), and one Actinopterygii (*Danio rerio*) by neighbor-joining method using the Poisson model by MEGA 6.0 with 1,000 bootstraps (The Biodesign Institute, Tempe, AZ).

### Immunofluorescence

For OCT frozen samples, 10-μm-thick sections were stained for β cells using guinea pig anti-insulin antibody (DAKO, A0564) and mouse anti-Urocortin 3 antibody (Santa, sc-517449), for α cells using mouse anti-glucagon antibody (Sigma G2654), and for δ cells using rat anti-somatostatin antibody (Abcam ab30788), followed by various Alexa Fluor-conjugated goat secondary antibodies (Jackson immunoresearch laboratories or molecular probes). We used confocal microscopy and Laser Scanning Microscope Software (Leica TCS SP8 STED) to survey colocalization and capture images.

### Immunohistochemistry

Fresh frozen sections from pygmy sperm whale pancreas, 10 μm in thickness, were pretreated with hydrogen peroxide, 1% Triton X-100 in PBS, 0.1 M glycine, and blocking buffer (5%FBS and 0.1% tween-20 in PBS). Pancreas sections were incubated with mouse anti-insulin antibody (Beyotime, AF0204) and/or mouse anti-glucagon antibody (Sigma G2654). After primary antibody treatment, the sections were incubated with horseradish peroxidase-conjugated secondary antibody (ZSGB-BIO, PV-9000) for 1 h. Positive reactions were visualized with diaminobenzidine (ZSGB-BIO, ZLI-9018), and the sections were counterstained with hematoxylin.

### Hematoxylin and Eosin (H&E) Staining

Fresh frozen sections from the pygmy sperm whale pancreas were treated with 4% paraformaldehyde-PBS, and hydrated in distilled water, stained with hematoxylin (1 min), differentiated in 1% hydrochloric acid alcohol, blued in ammonia water, counterstained with eosin (30 s), dehydrated with ethanol at different concentrations (70, 80, and 95% ethanol, anhydrous ethanol), transparentized with xylene, and finally mounted in neutral gum.

### Quantification of Islet Area, Statistics of the Composition of Endocrine Cells

Quantifications of stained areas were performed on digital images using the imageJ software. This software was programmed to automatically quantify stained areas within defined regions of interest. The insulin plus glucagon staining sections, insulin staining section, and glucagon staining section were measured for islet, β cells and α cells areas, respectively. Each measurement was average from 5 difference sections and expressed as the mean ± S.E.M. The percentage of the endocrine cell area were calculated as the specific endocrine cell area compared with the total pancreas area. For the proportions of endocrine cells, 11 islets were counted based on 5 difference immunofluorescence staining sections, pancreas regions were randomly selected and hormone positive endocrine cells with different fluorescent colors were counted.

## Results

### Molecular Identification of the Specimen

The toothed whale specimen was measured 2.73 m and suggested to be a female pygmy sperm whale (*K. breviceps*) or a dwarf sperm whale (*K. simus*), based upon the external physical and morphological examination (Supplemental Figure 1A). To further identify the species, we successfully amplified and sequenced for the three mitochondrial regions from genomic DNA, the 5′ end of the *cox1* gene (636 bp), the partial *cytb* gene (574 bp), and the complete D-loop sequence (938 bp). The *cox1* sequence submitted to the BOLD Systems matched to pygmy sperm whale reference sequences with 100% similarity (Supplemental Figure 1B). The DNA Surveillance analysis results showed the present specimen sequences clustered with pygmy sperm whale reference sequences for both D-loop (Supplemental Figure 1C) and the *cytb* (Supplemental Figure 1D) with high bootstrap support (100%). According to literature (16) and our anatomical observation, as well as the molecular information, we identified that the whale was an adult female pygmy sperm whale (*K. breviceps*).

### Identification and Characterization of Pygmy Sperm Whale Insulin

Two exons and one intron in the pygmy sperm whale *insulin* gene locus were obtained from genomic DNA, which contained the CDS of 333 bp encoding a 110 amino acid preproinsulin protein (Figure 1A). The nucleotide and deduced amino acid sequence has been deposited in GenBank (accession no. MN581742). The preproinsulin contains signal peptide of (residues 1~24), B-chain (residues 25~54), C-peptide (residues 55~89), and A-chain (residues 90~110) (Figure 1B). Overall, preproinsulin was highly conserved compared with other mammalian orthologs (Figure 1B). The identity of pygmy sperm whale preproinsulin with other vertebrates preproinsulin orthologs was 93.6% (Common bottlenose dolphin), 93.6% (Finless porpoise), 88.2% (Pig), 87.3% (Human), 86.4% (Bovine), 79.1% (Mouse), 62.8% (Chicken), 59.3% (Chinese Alligator), 53.9% (Tropical clawed frog), and 43.1% (Zebrafish). The mature pygmy sperm whale insulin peptide, A-chain and B-chain, was highly conserved. The greatest sequence identity was observed in the B-chain, being 84.0~100% identical among different species, with the identity in the A-chain at 66.7~100%. However, the C-peptide was more variable among different vertebrate preproinsulin orthologs.

**Figure 2:**
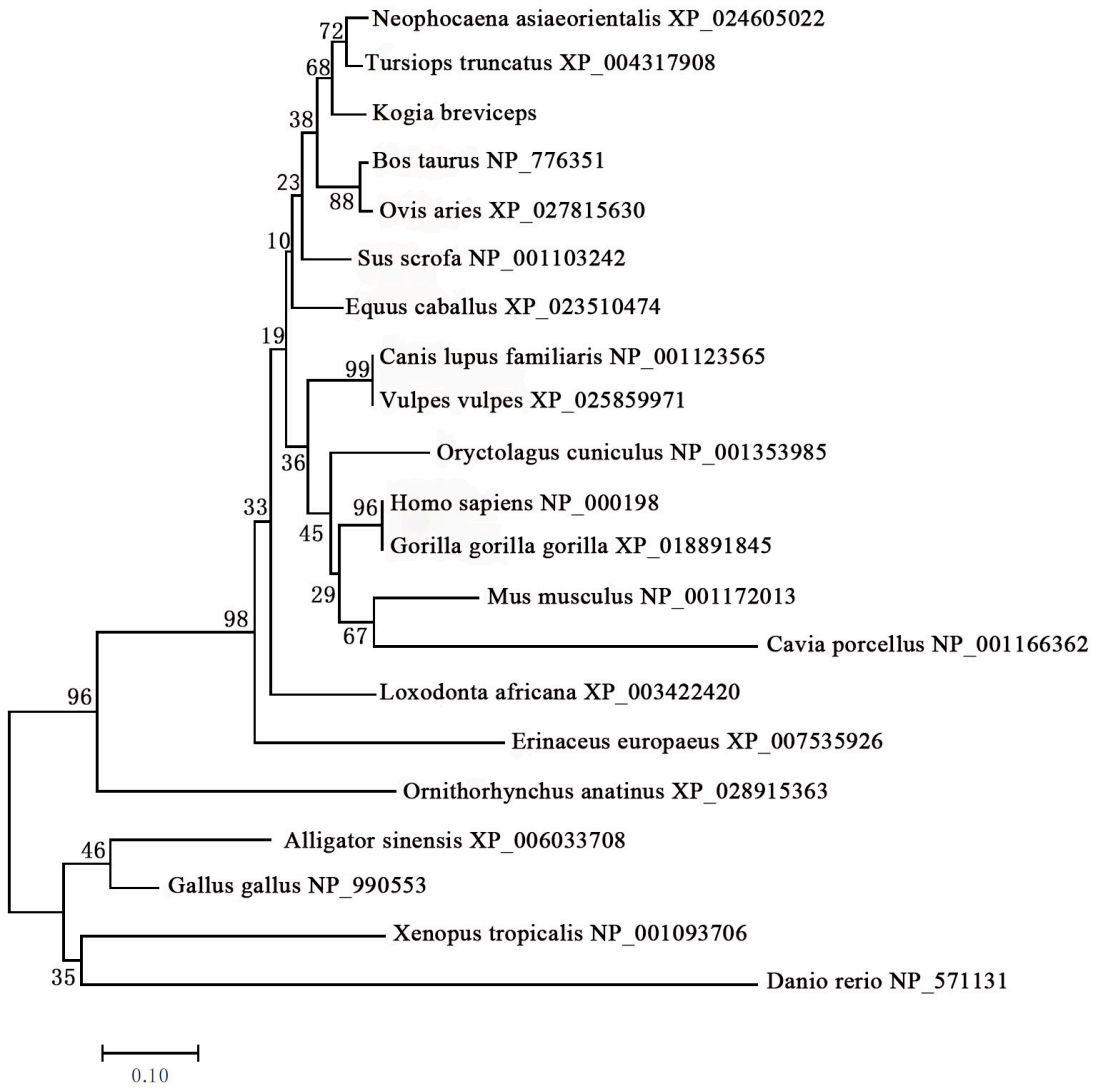
Phylogenetic analysis of the preproinsulin gene family. Full-length sequences of preproinsulin were analyzed using the neighbor-joining method. Numbers on nodes represent the frequency with which the node is recovered per 100 bootstrap replications in a total of 1,000.

We next performed phylogenetic analysis using the sequences of 21 vertebrates preproinsulin obtained from GenBank. A phylogenetic tree generated by the neighbor-joining method revealed that the pygmy sperm whale preproinsulin forms a cluster with the cetacean predicted preproinsulin [Finless porpoise (*N. asiaeorientalis*) and Common bottlenose dolphin (*T. truncatus*)] with high bootstrap support value, suggesting that the protein was indeed the ortholog of the cetacean preproinsulin (Figure 2).

### Identification and Characterization of Pygmy Sperm Whale Glucagon

**Figure 3:**
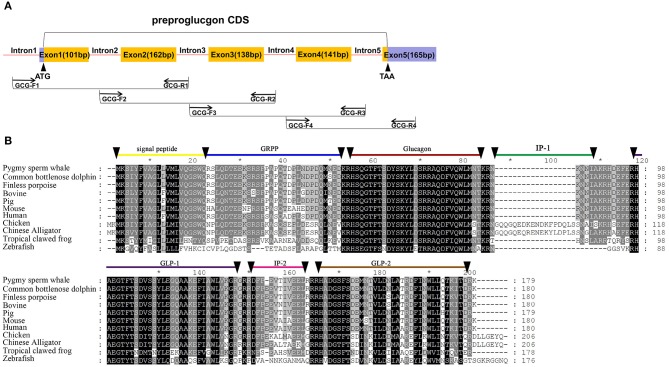
Identification of *preproinsulin* from pygmy sperm whale. **(A)** Schematic representation of the PCR strategy to clone *preproglucagon*. **(B)** Sequence comparison of vertebrate preproglucagon primary sequences. Sequence alignments were obtained with Genedoc by the Clustal method. The signal peptide (residues 1~20), GRPP (residues 21~50), glucagon (53~81), IP-1 (residues 84~89), GLP-1(residues 98~127/8) IP-2 (residues 131~143), and GLP-2 (residues 146~178) are labeled.

Five exons and four introns of the *glucagon* gene were obtained from pygmy sperm whale genomic DNA, which contained the CDS of 540 bp encoding the full-length preproglucagon of 179 aa (Figure 3A). The nucleotide and deduced amino acid sequence has been deposited in GenBank (accession no. MN581743). The preproglucagon aa sequences showed a high sequence similarity compared to different species (Figure 3B). The similarity of pygmy sperm whale preproglucagon with other preproglucagon vertebrate orthologs was 98.3% (Common bottlenose dolphin), 97.8% (Finless porpoise), 96.1% (Pig), 92.8% (Human), 94.4% (Bovine), 87.7% (Mouse), 63.1% (Chicken), 61.7% (Chinese Alligator), 54.5% (Tropical clawed frog), and 39.6% (Zebrafish). The mature glucagon peptide (residues 53~81) and GLP-1 (98–127) sequence was particularly conserved, showing 100% matching in mammalian orthologs (Figure 3B).

**Figure 4:**
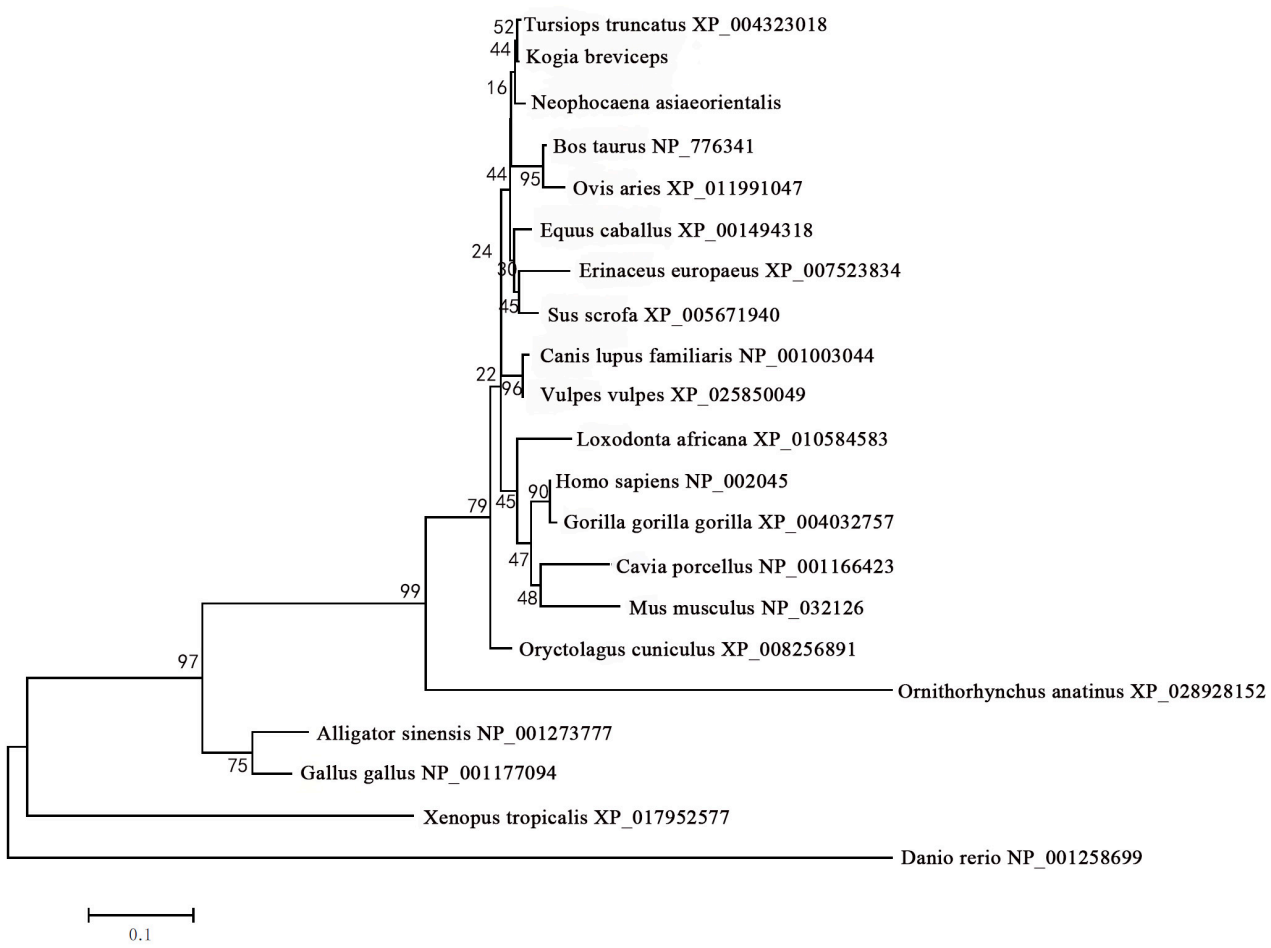
Phylogenetic analysis of the glucagon gene family. Full-length sequences of preproglucagon were analyzed using the neighbor-joining method. Numbers on the nodes represent frequency with which the node is recovered per 100 bootstrap replications in a total of 1,000.

The phylogenetic tree generated by the neighbor-joining method using 21 vertebrates preproglucagon revealed that the pygmy sperm whale preproglucagon forms a cluster with the cetacean predicted preproinsulins [Finless porpoise (*N. asiaeorientalis*) and Common bottlenose dolphin (*T. truncatus*)], The closest match was with the Common bottlenose dolphin with high bootstrap support value, suggesting that this protein is indeed the ortholog of the cetacean preproglucagon (Figure 4).

### Pygmy Sperm Whale Islet Distribution

**Figure 5:**
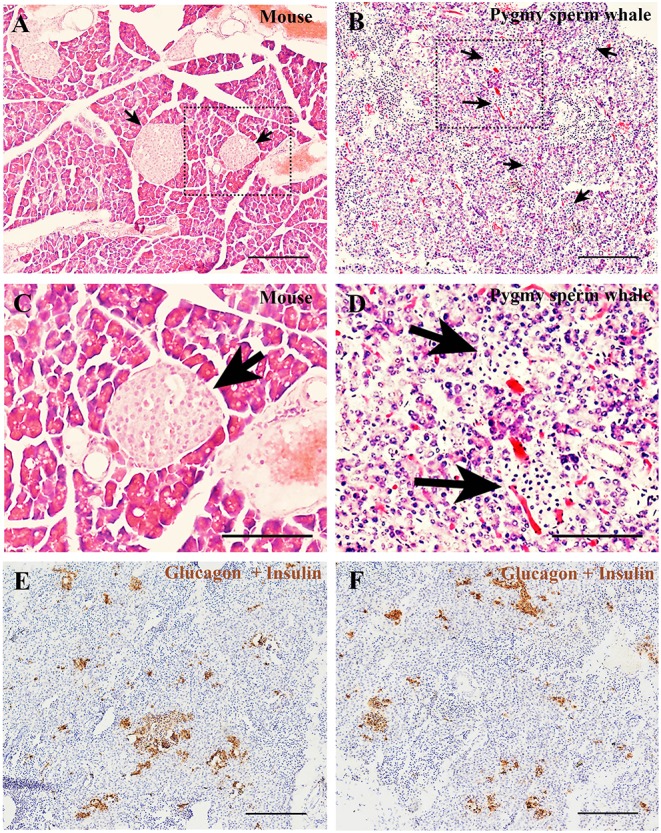
Pygmy sperm whale islet distribution. **(A,B)** Hematoxylin and eosin stained section of pancreas from mouse **(A)** or pygmy sperm whale **(B)** Arrows indicated the islet in each species, scale bars are the same which indicate 200 μm. **(C,D)** Magnification images of islets from dashed rectangle boxes from **(A**, **B)** respectively, scale bars indicate 150 μm. **(E,F)** Two representative images immunohistochemistry co-stained section of pygmy sperm whale pancreas with both glucagon and insulin antibodies. Scale bars indicate 200 μm.

**Figure 6:**
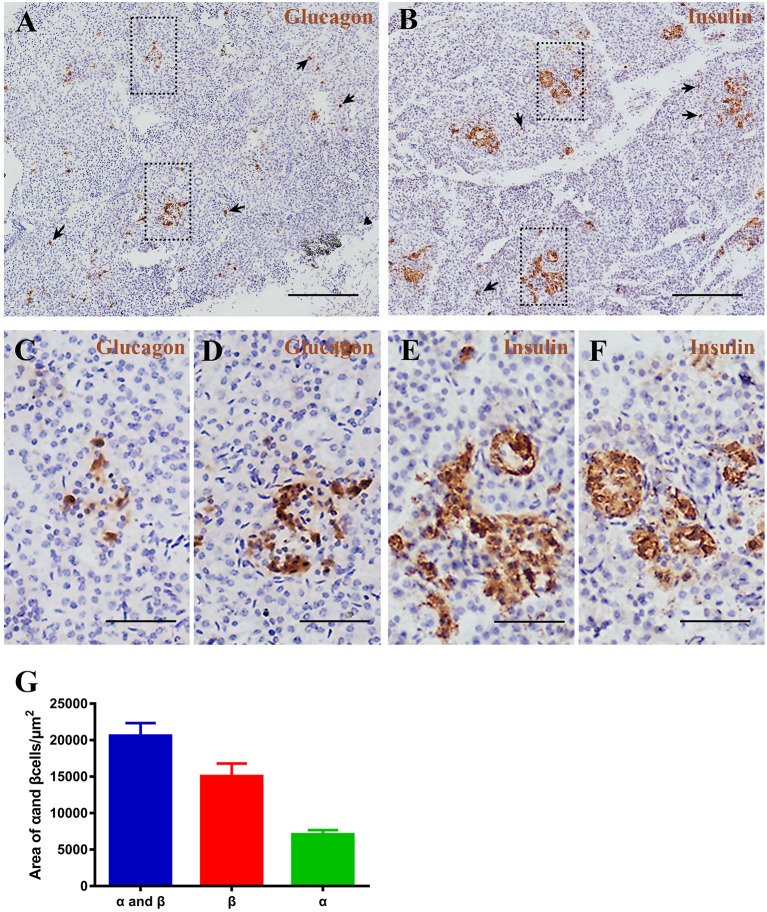
Pygmy sperm whale islet α cells and β cells distribution. **(A,B)** Representative, images of pygmy sperm whale pancreas stained by single immunohistochemistry. α-cells **(A)** stained with antibody against glucagon protein. β-cells **(B)** stained with antibody against insulin protein. The arrow indicated the small clumps or single endocrine cells scattered throughout the pancreas. Scale bars indicate 200 μm. **(C,D)** Magnification images of α cells from dashed rectangle boxes from **(A)** scale bars indicated 50 μm. **(E,F)** Magnification images of β cells from dashed rectangle boxes from **(B)** scale bars indicated 50 μm. **(G)** Summary graph of α cells, β cells, and α cells plus β cells areas from sections.

To determine the islet distribution in the pancreas, we first performed hematoxylin and eosin staining (H&E) on the pygmy sperm whale pancreas sections. We identified two different types of parenchymal tissue, the dark-stained acinar cells of the exocrine pancreas, and the light-stained islets of Langerhans (Figures 5B,D). Overall, islet shapes were irregular and scattered throughout the pancreatic lobules. Pygmy sperm whale islets were smaller in size and the border of acinar cell and islet was more obscure (Figure 5B), compared to mouse pancreatic islets (Figures 5A,C). Considering the difficulty in defining the islet area in the pygmy sperm whale pancreas by H&E, we also performed immunohistochemistry using both insulin and glucagon antibodies (Figures 5E,F). Insulin and glucagon co-staining confirmed the pancreatic islets in the pygmy sperm whale were irregular in shape, with only few oval in shape, unlike islets in both mouse and human. Furthermore, islet size was assorted, as demonstrated on both insulin and glucagon co-stained sections, the islet area of pygmy sperm whale was 20608 ± 1726 μm^2^, with 1.41 ± 0.35% of total pancreas area (Figure 6G, blue bar).

### Pygmy Sperm Whale Islet Architecture and Composition

To investigate pancreatic islets in the pygmy sperm whale further, we assessed the morphology of glucagon-secreting α cells and insulin-producing β cells. First, we performed immunohistochemistry using glucagon or insulin antibodies, separately. Pygmy sperm whale α and β cells were clustered randomly throughout the pancreatic lobes with various shapes (Figures 6A–F). Additionally, few glucagon-positive α cells or insulin-positive β cells without islet outlines were scattered throughout the exocrine pancreatic tissue (Figures 6A,B arrows). For α cells, as a percentage of total pancreas area was 0.47 ± 0.07%, with the area of 7,085 ± 589.9 μm^2^ (Figure 6G, green bar). The insulin positive β cells, as a percentage of total pancreas was 0.99 ± 0.22%, with the area of 15,073 ± 1,725 μm^2^ (Figure 6G, red bar).

**Figure 7:**
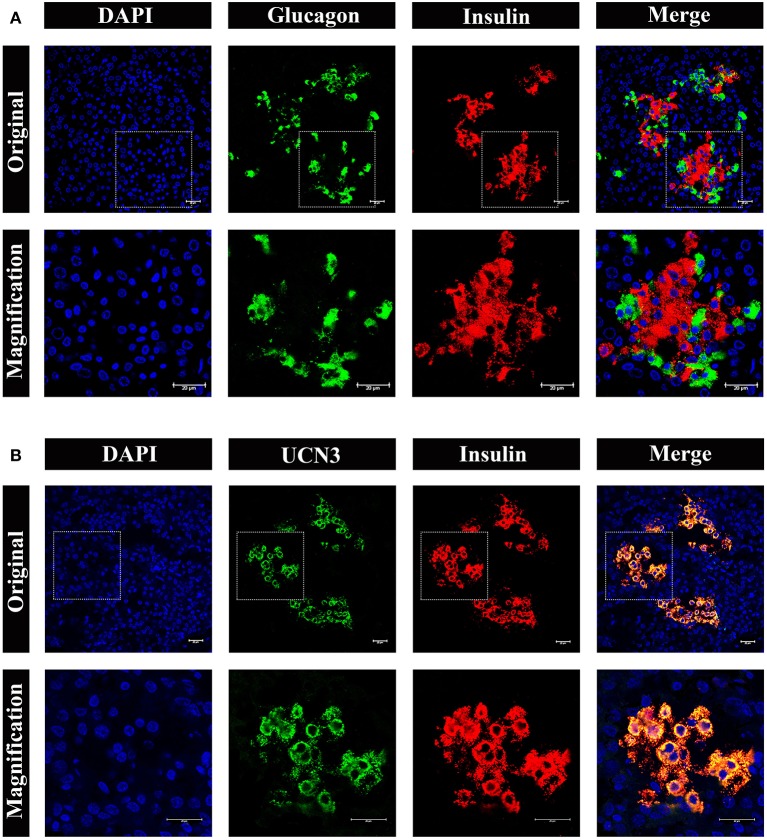
Architecture of α cells and β cells in pygmy sperm whale islet. **(A)** Representative, confocal images of pygmy sperm whale pancreatic α cells and β cells by immunofluorescence staining. The nuclei are shown with DAPI (blue), the α cells are labeled by an antibody against glucagon protein (green), and the β cells are indicated by the antibody against insulin protein (red). **(B)** Representative, confocal images of UCN3 staining. UCN3 was labeled by an antibody against UCN protein (green), β cells are indicated by the antibody against insulin protein (red). For **(A,B)** upper panel original images were taken by the 63X lens, and the lower panel is the amplification images, shown in the dashed squares. Scale bars indicated 20 μm.

To obtain further information on the distribution and composition of endocrine cells in the islet, we performed multi-color immunostaining using antibodies against glucagon and insulin. As shown in Figure 7A, the majority of islets displayed β cells in the islet core, with glucagon positive α cells in the islet periphery. However, we also observed a small number of α cells scattered throughout the islets, including the center of the islet core (Figure 7A, bottom panel). Considering the urocortin 3 (UCN3) transcription factor regulates both human and mouse β-cell maturation, we also examined UCN3 immunostaining in pygmy sperm whale (Figure 7B). This mature β-cell marker showed a high degree of uniformity when co-stained with insulin in the pygmy sperm whale islets, with the subcellular location in the cytoplasm of the β cells, as described in human and mouse (17, 18).

**Figure 8:**
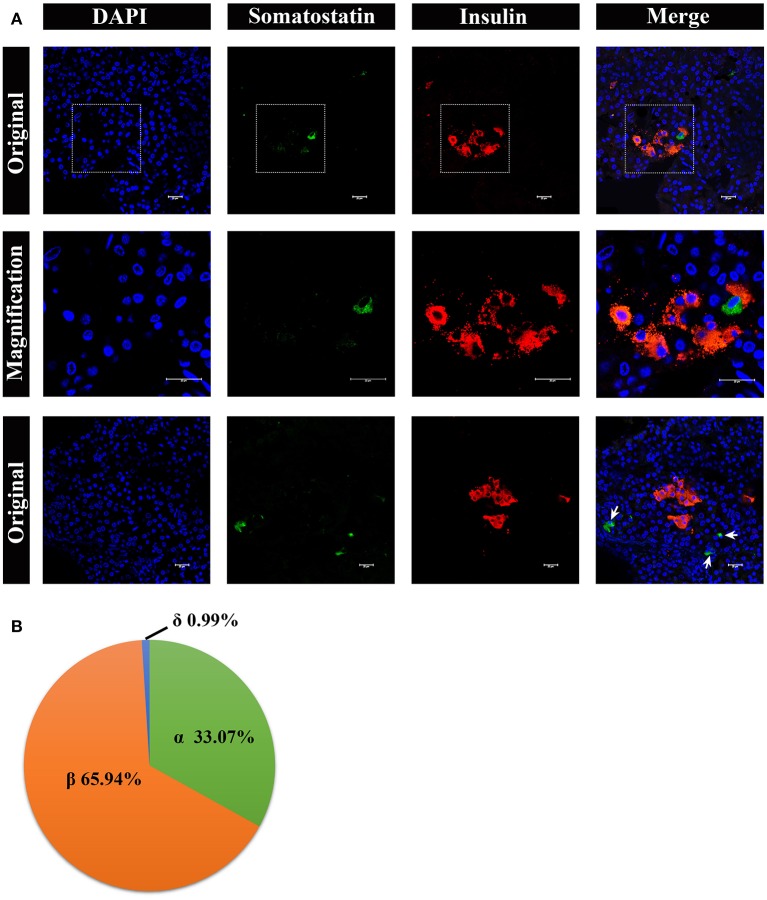
Architecture of δ cells and β cells in pygmy sperm whale islet. **(A)** Representative, confocal images of pygmy sperm whale pancreatic δ cells and β cells by immunofluorescence staining. The nuclei are shown with DAPI (blue), the δ cells were labeled by an antibody against somatostatin protein (green), and the β cells were indicated by the antibody against insulin protein (red). The upper panel is the original images taken by the 63X lens, the middle panel is the magnification images shown in the dashed squares, and the lower panel are the images to show some δ-cells (arrow) scattered throughout the pancreas, together with β cells. Scale bars indicated 20 μm. **(B)** The pie charts show the proportions of the α cells, β cells, and δ cells in pygmy sperm whale islets.

Lastly, we analyzed somatostatin-positive δ cells, by immunostaining, shown in Figure 8. The δ cells were very few in number in the pancreatic islets, surrounding the β cells (Figure 8A, middle panel). However, there were also a few δ cells scattered in pancreas lobes alone (Figure 8A, lower panel). The percentage of α cells, β cells, and δ cells in the pancreatic islets were ~33.07%, 65.94%, and 0.99%, respectively (Figure 8B).

## Discussion

The islets of Langerhans are comprised of highly specialized endocrine cell populations, including α cells, β cells, and δ cells (19). Both insulin and glucagon have been of significant focus, due to the key roles in glucose metabolism, diabetes and other disorders (20). Importantly, the genetic sequences of both hormones found in cetaceans have yet to be identified, however we now, for the first time, report both insulin and glucagon cetacean sequences.

In this study, we cloned and characterized both *insulin* and *glucagon* genes from the pygmy sperm whale. To our knowledge, this is the first time the full-length structure of *preproinsulin* and *preproglucagon* have been determined in a cetacean species. By phylogenetic analysis, we confirmed that the gene we cloned was pygmy sperm whale *preproinsulin* (Figure 2). The preproinsulin protein was highly conserved in pygmy sperm whale, compared with other vertebrates. The mature insulin peptide (B-chain and A-chain) was 100% identical in the cetacean species, also observed in the pig (*Sus scrofa*), belonging to artiodactyla group (21), and only have a few amino acid differences with other mammalian insulin sequences (Figure 1B).

For the pygmy sperm whale, the amino acid sequence of preproglucagon was highly similar to the other mammalian orthologs, and exhibited a highly conserved region which encodes mature glucagon peptide and GLP-1 peptide and showed 100% identity with human, mouse, pig and bovine sequences (Figure 3B). The insulin and glucagon molecular network regulates many key biological processes of organisms, including reproduction, development, metabolism, and lifespan (19, 22, 23). Our results showed that both preproinsulin and preproglucagon proteins of pygmy sperm whale were highly similar to mammals and may suggest that the function of both hormones is also conserved in cetaceans, including pygmy sperm whale. However, further investigation is needed to determine differences in pre and post-translational modifications and the epigenetic regulation of both proteins in pygmy sperm whale, compared to other mammals.

So far, histomorphology of endocrine pancreas has been analyzed in several different species of cetaceans. Such as, area and architecture of endocrine cells were evaluated from 22 bottlenose dolphin (*Tursiops truncatus*), and the islet area was positive linear association with dolphin age (24). Using Peroxidase-antiperoxidase (PAP) techniques, the location of α cell and β cell were showed in the pygmy sperm whale (*K. breviceps*) and dwarf sperm whale (*Kogia simus*) (25). Moreover, the pancreas weight, islet size, and distribution of α cells and β cells were also investigated in the beluga whale (*Delphinapterus leucas*) (26). However, more detailed analyses are still required to understand the physiological study in cetaceans. Therefore, we combined H&E, immunohistochemistry and immunofluorescence techniques to study pygmy sperm whale islet distribution and architecture. Our results showed that the pygmy sperm whale islets are randomly distributed, but clustered within the pancreatic lobes in various shapes and sizes (Figure 5). The mean islet size was smaller than that of the mouse (Figure 5), however the islet volume was similar to other mammals, particularly to the bottlenose dolphin and pig (24, 27, 28). Surprisingly, the architecture was variable across the different species analyzed (29). Specifically, mice islets have a β-cell-rich core which is surrounded by few α cells and δ cells. While in human islets, the α cells, β cells, and δ cells appear to be randomly distributed throughout the islet (28, 29). In pygmy sperm whale islet, β cells were in the central core, the majority of α cells in the periphery and a few α cells dispersed throughout the islets (Figures 6, 7). This architecture is somehow between mice and human, but more close to mice than humans. In bottlenose dolphin, β cells were found clustered in the cords, while α cells were found both dispersed or in the periphery (24). These arrangements of α cell and β cell in pygmy sperm whale and bottlenose dolphin are highly similar to their closest evolutionary relatives, animals in the Artiodactyla. The islet architectures of pigs (*Sus domesticus*), sheep (*Ovis aries*), and cow (*Bos taurus*) all display β cells centrally located, and most α cell reside in the periphery, along with some α cells are located in the center of islets (27, 29–33). Taken together, these studies may reveal that the animals in the Artiodactyla have preserve highly similar islet architecture during evolution. However, in previous studies of beluga whale and pygmy sperm whales suggested that β cells centrally located in the islets and the α cells are peripherally. These difference from early studies may due to the individual difference or low resolution of previous staining techniques (25, 26).

In addition, individual endocrine cells such as α, β, and δ cells can be observed outside the islets (Figures 6–8). This phenomenon was also detected in and many other mammalian species, including rat, cat, dog, pig, non-human primate, bottlenose dolphin, and beluga whale (24, 26, 34, 35). Although why this kind of endocrine cell arrangements in these species are still largely unknown, some studies suggested that these alone endocrine cells may be an indication of proliferation or neogenesis (34–37). The composition of α and β cells were 33.07% and 65.94%, respectively, in pygmy sperm whale islet, resembling the human islet arrangement (20–40% α-cells and 50–70% β-cells) (1).

In summary, we have identified *insulin* and *glucagon* genes from the pygmy sperm whale, which is the first report about these two genes in cetaceans. We have further characterized α cells and β cells, including their distribution, architecture, and composition in pygmy sperm whale pancreas. These results provide new information about the structural conservation of the *insulin* and *glucagon* genes, and new information of the properties on endocrine cells in cetacean for further studies. Although the lifestyle and nutrition state were changed during evolution, he highly conserved genes sequences and islet location of insulin and glucagon hormones, suggested that cetaceans preserve the insulin and glucagon physiological function during the adaption from terrestrial to fully aquatic environment.

## Data Availability Statement

The datasets generated for this study can be found in the Genbank (MN581742 and MN581743).

## Ethics Statement

This animal study was reviewed and approved by Xiamen University Institutional Animal Care and Use Committee.

## Author Contributions

ML and XW was the guarantor of this work and as such, had full access to all of the data in the study, takes responsibility for the integrity of the data, and the accuracy of the data analysis. ML, LZ, and XW designed the study. LZ, LW, and RA performed the key experiments and drafted the manuscript. LZ, LW, RA, WW, XW, and ML participated in the planning of the work and the interpretation of the results. ML, XW, and WW have participated in the revising of the paper.

### Conflict of Interest

The authors declare that the research was conducted in the absence of any commercial or financial relationships that could be construed as a potential conflict of interest.

## Supplementary Material

Supplemental Figure 1 Morphological and molecular identification of pygmy sperm whale. **(A)** External appearance of the toothed whale suggested it is a female pygmy sperm whale (*K. breviceps*) or a dwarf sperm whale (*K. simus*). **(B)** Based on global database of cox1 sequences, the BOLD Systems gave a NJ tree showing the relationship of *cox1* sequence from the sequences we obtained in this study (present sequences with red arrow) to its 100 nearest neighbors. The present sequences showed 100% sequence similarity with the pygmy sperm whale reference sequences. **(C)** NJ tree based on *D-loop* query and reference sequences of all recognized cetaceans. **(D)** NJ tree based on *cytochrome b* query and reference sequences of all recognized cetaceans. Bootstrap values based on 1,000 replicates. Both DNA surveillance trees showing high bootstrap support (100%) grouping “present sequence” we obtained in this study (with red arrow) with the reference sequences of pygmy sperm whale.
