# Supplementary figures and images for: Characterization of Insulin and Glucagon Genes and Their Producing Endocrine Cells From Pygmy Sperm Whale (Kogia breviceps)
Source: Front Endocrinol (Lausanne). 2020 Mar 31;11:174. doi: 10.3389/fendo.2020.00174 (PMC7137828; doi:10.3389/fendo.2020.00174)

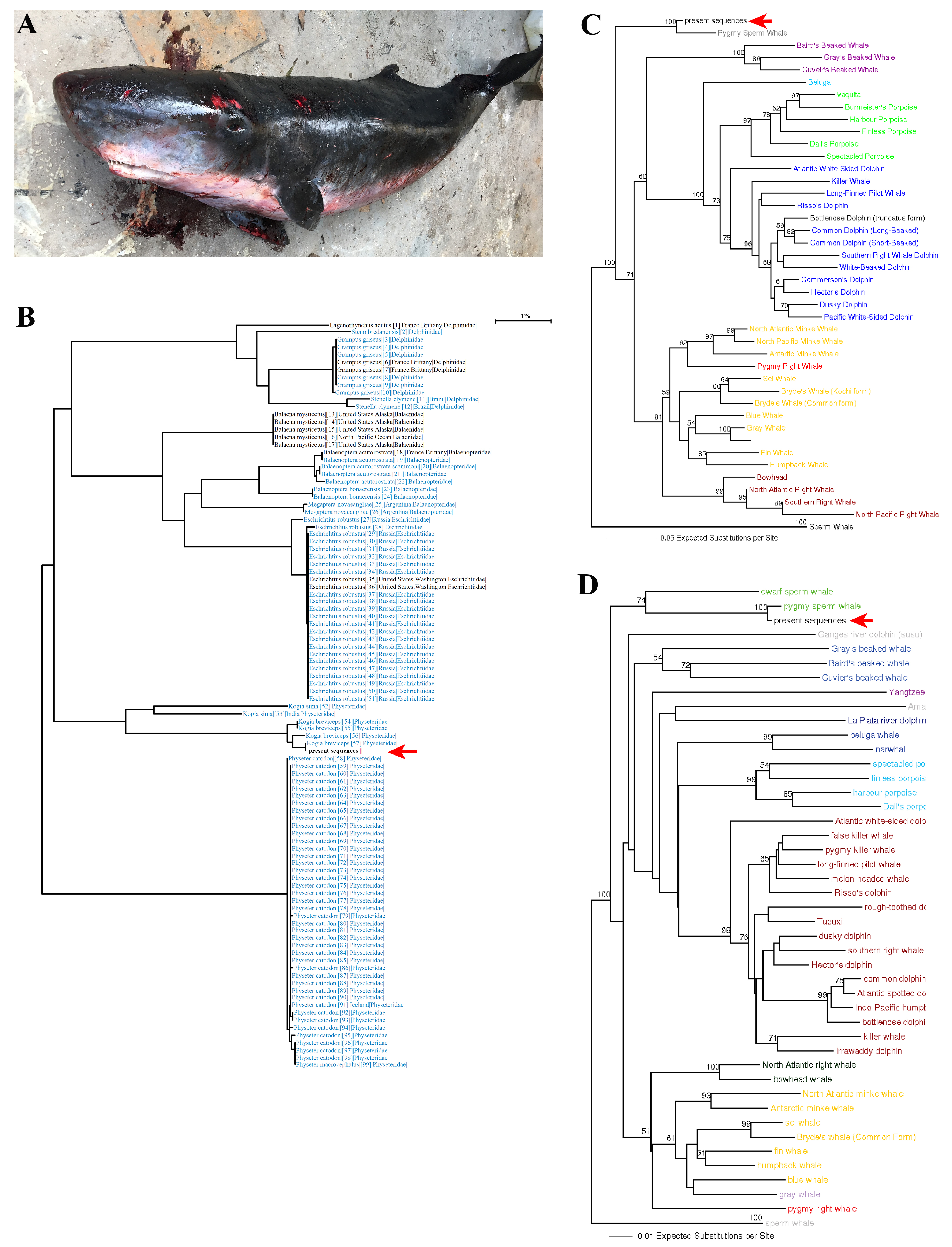

Supplement: Supplemental Figure 1 — Morphological and molecular identification of pygmy sperm whale. (A) External appearance of the toothed whale suggested it is a female pygmy sperm whale (K. breviceps) or a dwarf sperm whale (K. simus). (B) Based on global database of cox1 sequences, the BOLD Systems gave a NJ tree showing the relationship of cox1 sequence from the sequences we obtained in this study (present sequences with red arrow) to its 100 nearest neighbors. The present sequences showed 100% sequence similarity with the pygmy sperm whale reference sequences. (C) NJ tree based on D-loop query and reference sequences of all recognized cetaceans. (D) NJ tree based on cytochrome b query and reference sequences of all recognized cetaceans. Bootstrap values based on 1,000 replicates. Both DNA surveillance trees showing high bootstrap support (100%) grouping “present sequence” we obtained in this study (with red arrow) with the reference sequences of pygmy sperm whale. [file Image_1.JPEG]
